# The Iterative Development of Medicines Through the European Medicine Agency's Adaptive Pathway Approach

**DOI:** 10.3389/fmed.2019.00148

**Published:** 2019-06-27

**Authors:** Giuseppe Nicotera, Gianluca Sferrazza, Annalucia Serafino, Pasquale Pierimarchi

**Affiliations:** Institute of Translational Pharmacology, Department of Biomedical Sciences, National Research Council of Italy, Rome, Italy

**Keywords:** regulatory tools, European medicines agency, adaptive pathways, HTA bodies, iterative development

## Abstract

The development of novel regulatory tools such as adaptive clinical trial design and utilization of real-world evidence are topics of high interest. Recently, the European Medicines Agency (EMA) introduced the Adaptive Pathways (AP) that represents an innovative tool in healthcare systems allowing the early dialogue with multiple stakeholders on promising and innovative medicinal products in areas with an high unmet medical need. The innovative aspect in the AP is the early involvement of several stakeholders such as pharmaceutical industry, the Academia, Health Technology Assessment (HTA) bodies, and patient representatives bringing their real experience with the disease and their expectations about the treatment. AP is not a new licensing tool but an opportunity for a very early discussions, before starting the phase II studies, among all stakeholders, including regulators, companies, HTA bodies, and patient representatives on a new potential medicine in areas of high unmet medical need. The aim of this paper is to describe the evolution of the AP approach from the beginning of the pilot project to date, highlighting major advances, and achievement at European level.

## Introduction

The continuous progress in sciences and technologies has the potential to bring a wide range of beneficial therapies to patients over the coming years. These include personalized and precision medicine and advanced therapies that will require new modalities of evaluation and new ways of managing them in clinical practice^1^. Between the multiple roles of the EMA one of the most important is the coordination and the cooperation with the medicines regulatory authorities at national levels, in order to facilitate a dialogue and a wider exchange of information, thus strengthening the quality, safety, and efficacy of medicinal products globally. One of the main goals for all the Regulatory Medicine Agencies is to counterbalance the need to have timely access to promising medicines for all patients, without compromising patients' safety. Until now the concept and development of new drugs has a well-structured, defined and rigid process that require about more than 10 years for the research, development, and authorization and costs around USD 1 billion, where only 1% of medicines reach the market (1). Patients suffering from conditions with no available treatments rarely have time to wait decades for a new drug to come to market. Recent developments are promoting the transition from the traditional approach, which involves extensive testing and the marketing authorization for large groups of patients, to an adaptive approach, characterized by the development of innovative clinical trial setting that could comprise a greater involvement of multiple stakeholders in the active decision-making process (2). During the past years EMA developed several new processes and tools in order to have an earlier access of new promising medicines especially in areas of high unmet medical needs and for orphan diseases. These early access and registration tools includes scientific advice/protocol assistance, Priority Medicine (PRIME), conditional marketing authorization and approval under exceptional circumstances, patient registries, accelerated assessment, pre-submission meeting, post-authorization measures, post-approval change management protocol, parallel EMA-HTA scientific advice, and compassionate use. Differently from afore mentioned early registration tools, AP is not a new licensing pathway because does not allow an early access but is a new approach in the development of new medicines based on the early dialogues among relevant stakeholders. The AP allow a new way of getting clinical data in order to design a smart development program that acquires the relevant evidence base, using all data sources, for a seamless decision making transition. This includes better ways to acquire data compared to the traditional Randomized Controlled Trials (RCT) setting, using the well-established European regulatory framework and without changing the standards for the evaluation of benefits and risks (3).

The AP was initially termed and proposed by the EMA as “adaptive licensing.” Since then the concept has been renamed AP in order to make a correct focus on the development and the iterative development and introduction of medicines to the market, rather than a new way for regulating and authorizing medicines earlier (4). It is desirable, in the future, the inclusion of a systematic, early dialogue, and of multiple stakeholders in all medical developments that have reached certain milestones and are promising (3). Despite the fact that AP has met with considerable interest, there is still wariness about the new development schemes and some discussants report that is a difficult concept to convey to stakeholders. Given the distrust of the conservative class, one needs to wonder whether such iterative schemes will be able to determine the time needed for drug approval and how such schemes will allow a real change in market access modalities, especially at national and regional level. Following the approach of the APs, it is desirable that, on the one hand, there is an ever greater level of transparency between industry and decision-makers and, among them, and patients who use the new drug. On the other hand, it is also essential to have greater involvement of regulatory authorities in the risk monitoring and mitigation phase from initial licensing, including through the most widespread implementation of registers, the use of data collected through electronic medical records and the development of post-authorization observational studies.

## The Principles Behind the AP Approach

AP is based on three practical principles that focus the attention on the (1) development of an active early dialogue with regulators (before the beginning of phase II studies), HTA bodies, patient, and healthcare professional representatives for a joint comparison on the clinical development programs prior to the assessment of large confirmatory trials; (2) the use of real-world data to supplement clinical trial data on the basis of an iterative development, which means initial focus on a narrow and; (3) a well-defined patient population with the perspective planning of possible expansion of the indication to other patients (5). Compared to the traditional procedure, the AP is an incremental process by which the Marketing Authorization (MA) of the new product, its price and the conditions of eligibility may be discussed again in time based on new evidence. Specifically, the company shares and agrees with the Agency a development protocol of the efficacy/effectiveness data collected in actual practice (real world evidence) to amend (increase or decrease) over time the access mechanisms and criteria and then eventually define a full marketing approval. Conversely, based on available regulatory tools and processes, the AP approach proposes several innovative solutions such as effectiveness studies, predictive methodologies, the active control of prescriptions in order to mitigate off-label use in the initial market access phases, with an emphasis on post-authorization real-world evidence, which requires substantial efforts and capacity building for collection and data analysis.

From this point of view the “heart” of the AP approach is represented by the trade-off between accessibility and uncertainty (6).

### Early Access and Regulatory Tools in the European Framework

As mentioned before the AP approach is based on an iterative development that builds on already existing regulatory tools in the context of EU legal framework. These processes, early access and regulatory tools includes scientific advice/protocol assistance; compassionate use; the conditional approval, approval under exceptional circumstances, patient registries, and other pharmacovigilance tools that allow collection of real-life data and development of the risk-management plan for each medicine^2^. These aspects are essential in order to correctly differentiate all the early access tools from the concept of the AP. The latter was specifically built in order to made possible the early participation and dialogue of all relevant stakeholders in the regulatory and development process with the goal to establish a better way to collect and acquire useful data for the appropriate evaluation of the benefit-risk ratio of new promising drugs for which there is a high medical need. Here we briefly describes different early access tools and procedures highlighting the fact that AP is a new approach that focus on the need to have an earlier exchange of views between stakeholders and regulators that could lead to an earlier access if properly adopted.

**Scientific advice** is a procedure provided by EMA in order to support companies on the appropriateness of studies in the context of the development of a medicine. This tool has proved very useful for the availability of high-quality, effective, and acceptably safe medicines, for the benefit of patients. For the conduction of Scientific advice there is a specific multidisciplinary group of expert coming from others EMA's Committee called the Scientific Advice Working Party (SAWP). Scientific advice is a procedure that could be requested at any stage of development of a medicine eligible for centralized authorization procedure. It is known that, following the Agency's advice, the applicants increase the probability of a positive outcome. This is due to the nature of the procedure itself, because SA can ensure to the developers the conduction of the appropriate tests and studies on the basis of the indications coming from a multidisciplinary group of experts specifically designed for the field. Is important to clarify that SA is not a pre-evaluation of data but it focus on the development strategy planned for a specific medicine, is not legally binding and can't ensure a marketing-authorization. Protocol assistance is a particular form of scientific advice, specifically designed for orphan medicines for rare diseases. This kind of procedure includes other answers related to several criteria on the authorization of an orphan medicine that includes evaluation of significant benefit, similarity, and clinical superiority^3^. The European Medicines Agency (EMA) offers to drug developers the possibility to establish advice in parallel with the European Network for Health Technology Assessment (EUnetHTA) in order to get feedback from HTA bodies on the marketing authorization and the reimbursement of new drugs. These consultations can take place before or after the product has been made available on the market. Interchanges between regulators and HTAs allow and facilitate an easier patient access to innovative drugs for the benefit of general public health. The main advantage of this procedure lies in the fact that it is the only possible moment of meeting between competent bodies EMA, EUnetHTA, and HTA bodies in which a series of strategies and meetings are set up to allow a close dialogue between health decision makers, patient representatives and health professionals so that, their opinions and experiences are incorporated into the discussions^4^. Moreover, the European Medicines Agency (EMA) together with the US Food and Drug Administration (FDA) have developed a parallel scientific counseling program (PSA). The goal of the PSA program is to provide a systematic mechanism for EMA assessors and FDA auditors in the simultaneous exchange of information with the sponsor on scientific issues during the development phase of new medicinal products (i.e., new drugs and human biology). These interactions allow an increase in the dialogue between the two agencies, providing a greater understanding of the decisions underlying the regulatory choices and avoiding redundant actions. These PSAs allow for greater harmonization between the procedures and rules for placing medicines on the market between EMA and FDA, avoiding as much as possible any disputes or different positions in the drug evaluation process^5^.

**Priority Medicine (PRIME)** scheme is an early access tool provided by EMA in 2016 that specifically refers to cutting edge medicines and was designed in order to support a faster development in the field of serious diseases with high unmet medical need. Like the AP, PRIME uses already existing regulatory tools like scientific advice and the accelerated assessment procedure with the same approach used in the field of innovative medicines that address an unmet medical need and could bring major therapeutic advantages to patients significantly improving their quality of life. Due to the voluntary nature of the scheme, if a candidate medicine is selected to be eligible for PRIME, the Agency offers several advantages like providing scientific advice, the involvement of multiple stakeholders and HTA bodies in order to accelerate the assessment and marketing authorization. An important element of this scheme regards that is driven principally by patients' needs, offering options in areas with no treatment available or additive therapeutic advantages over existing insufficient treatments, without reducing patient safety and sustaining an high level of evaluation standard. Is also an opportunity for developers in order to optimize and to expedite the development plan, facilitating the collection of reliable data, and a early patient access. Another aspect that particularly unites the AP to the PRIME scheme, regards the early dialogue that could ensure to the patients to participate in pragmatic trials designed to obtain the knowledge necessary for an application optimizing the best use of limited resources^6^.

**A conditional marketing authorization** is an early access tool provided by EMA when a company intends to support the development of a drug that meets the unmet medical needs of patients in the absence of all the scientific data usually required for marketing, only if it is demonstrated that the immediate availability of that medicinal product overcomes the risk of the absence of all scientific evidences requested. The eligibility is restricted to medicines that aim to treat, prevent or diagnose severely debilitating or potentially lethal diseases, including orphan drugs, where often, due to the lack of an adequate number of patients, it is not possible to conduct trials with high power and sample size. In exceptional cases and in emergency situations, less complete pharmaceutical, and non-clinical data may also be accepted. The conditional marketing authorizations can be granted only if the CHMP notes that the requirements regarding the risk-benefit ratio, the capacity of the applicant to provide complete data in the future, unmet medical needs; and when the public health benefit of the immediate availability of the medicine on the market overcomes the risks due to the need for further data, could be achieved. The holder will be required to complete specific obligations such us the conduction of new studies and/or additional activities with a view to providing comprehensive data confirming that the benefit-risk balance is positive. Conditional marketing authorizations are valid for 1 year and can be renewed annually. From 10 years to date, EMA granted 30 conditional marketing authorizations and until now, none had to be revoked or suspended. This data shows that conditional marketing authorization can help speed up patient access to new medicines^7^.

Another early access procedure is **the authorization under exceptional circumstances**. Differently from conditional authorization, the exceptional circumstances refer to the impossibility to achieve comprehensive data even after the authorization is granted. Due to the particular nature of this procedure is clear that its focus only on medicine where for the applicant is impossible to provide sufficient data on safety and efficacy under normal conditions of use; rare conditions or situation for witch the collection of a full set of information is impossible or unethical^8^.

**The Accelerated assessment** is another early access procedure that could ensure a reduction of the timeframe to review a marketing-authorization application. The Applications may be eligible for accelerated assessment if the CHMP decides that the product is a cutting edge medicine or regards an area of specific interest for public health and therapeutic innovation. Usually a standard centralized procedure can take up to 210 days excluding the timeframes of clock stops in which the applicant need to provide incremental evidences. The CHMP can reduce the timeframe to 150 days upon request if there are sufficient reasons for witch an accelerated assessment is suitable. Is highly recommended to request a pre-submission meeting with EMA in order to discuss the proposal with the CHMP and others responsible committees. This kind of procedure is suitable also for all those medicine that have been assessed as eligible for the access to the PRIME scheme. On the basis of afore mentioned requirements is clear that one of the point of major interest regards the potential therapeutic innovation of the medicines involved^9^.

### Early Dialogue With Multiple Stakeholders

The early dialogue with multiple stakeholders enables development plans of companies to include all relevant decision-makers involved rather than just the regulators (7). In the AP scenario several meetings are expected between stakeholders are held that are confidential and non-binding. This approach allows a wider group of stakeholders to put a critical eye on the clinical development pathway for the product by providing continue feedbacks on trials designs and on appropriateness of the selected endpoints, discussing evidence requirements for decision-makers, discussing ways to facilitate the earliest appropriate patient access, and exploring plans to reduce uncertainty after initial market introduction (4). Several organizations have been created to help the life sciences industry supply practical information about its products on the basis of real world evidence data. There have been several examples of informed patient associations established with the task to educate patient representatives and the lay public about in all the processes involved in medicines development.

One example is the “European Patients Academy on Therapeutic Innovation” (EUPATI) that addresses topics of interest in the field of regulatory science such as risk/benefit assessment, health economics, as well as patient involvement in drug development. The Project's objectives are to develop and disseminate accessible information and educational resources on therapeutic innovation for the establishment of expertise among well-informed patients (8). The establishment of an early dialogue is a great opportunity for HTA agencies and payers because it could ease the alignment between the evidence requirements of the various market access decision-makers and prescribers/patients. The early dialogue also promotes an efficient exchange of information between regulators and the harmonization of procedures such as national early access schemes, risk-sharing agreements, and others (9). In this way, already before that the medicine is authorized, all the stakeholders involved commit to carry out a legally bind plan of post-licensing development of knowledge generation for a medicine. The multi-stakeholder dialogue, together with the improvement of the use of innovative tools provided by the new pharmacovigilance systems, are two key elements in the AP scenario for the systematic monitoring of the safety and effectiveness of a medicine in clinical practice^10^. One good example of this trend is the recent approval of Biogen's nusinersen (Spinraza), the first treatment for Spinal Muscular Atrophy (SMA), thanks to the commitment of the patients and families who actively participated in the Spinraza clinical trial and the urgency demonstrated by the FDA in rapidly reviewing and approving this treatment (10). In the European scenario, the French Muscular Dystrophy Association (AFM-Téléthon) made several efforts in order to expedite the approval of this new treatment also in view of the extremely high expectations by the patients. Specifically, without waiting for the EMA's decision, AFM-Telethon invited Biogen to spread as more possible the use of France's regulatory framework in the field of early access to innovative drugs, i.e., temporary use authorizations (TuA), deemed to be one of the best in the world^11^.

### Real-World Evidence to Supplement Clinical Trial Data

The traditional clinical research process development often is not able to introduce and to face up to key issues of patients, physicians, and health systems on the appropriate role and use of new available and innovative treatments. The most typically unresolved issues include effectiveness, tolerability, and heterogeneity of treatment effects. Traditional efficacy studies tend to rigorously evaluate a single treatment. Differently, patients, suppliers and healthcare systems choose alternative treatments based on net benefit emerged in real practice. The evidence acquired from a real-world evidence setting often largely differ from the efficacy found in the standard clinical trial settings. Several Factors could contribute to enlarge this gap including the variation in practice settings, presence of comorbidities, patient adherence to the treatment, the use of other drugs and others. In the evaluation of primary outcome such as efficacy and safety data, these factors could be source of noise or biases. In daily clinical practice, these sources of change are directly relevant to patient and supplier decisions, and are crucial for those concerned who need to inform the decision-maker (11). The AP views drug development as an all-in-one process: all the stages of regulatory approval and evidence development need to run in parallel with marketing, and this implies that real world evidence (RWE) has been considered as a key component. In detail, The EMA proposes moving away from RCTs being used exclusively as the basis for regulatory decisions, and instead proposes using the entire toolbox of knowledge generation. This includes RWE data collection and studies in addition to conventional RCTs, pragmatic RCTs, and observational trials (12). At the same time great emphasis is placed on the need to reduce the levels of uncertainty of risk and the expected benefits of new drugs against faster access to new therapies. For this reason the essentials preconditions for the adaptive pathway approach are that should be initiated earlier, before the initiation of phase II studies (a substantial part of the refusals to get an AP is that industry asked for it in a later stage of the development) and the continued investment in the generation of real world evidence (RWE—for example, with the extensive use of pragmatic trial and observational studies) that could be considered complementary to the data of experimental studies (13). According to this approach, the new drug is subjected to several assessments and evaluations of the effectiveness during its whole life cycle (Life-span approach), instead of only into the phase of the pre-market access, allowing a reshaping of the value of the drug over the time. From this point of view, the AP represents a new paradigm of evaluation of the RWE and the EMA highlights how year-on-year advancements in RWE studies are seeing them becoming more systematic, generating increasingly reliable data, and undergoing improvements in methodology (12). Other important tools are the disease registries that could help to identify the natural history of a disease and which plays an important role for the place in therapy and for post-marketing surveillance of pharmaceuticals and the correct use of current standard of care (14). It is also important to reassess the validity of single arm studies in the context of orphan diseases and the comparison of the data obtained with outcomes inferred from disease registries (15). Other important actions regard the possibility to create a link between drug registries and risk-sharing schemes for reimbursement, the extension of clinical indication from a mixture of data obtained from disease registries and from compassionate use data and the assessment of post-authorization studies to investigate biomarker status of an all-comer population (16).

### Well-Defined Patient Population

Clinical studies are usually carried out on a sample of “ideal subjects” rather than whole populations. The most challenging aspect regards the identification of a random sample from the target population in which the results of the study would be generalized. In clinical practice, this aspect implies that some sampling bias occurs in almost all studies to a lesser or greater degree (17). The current approach is not designed in order to enable a timely and well-in formed patient access especially in the field of rare diseases. Often the criteria for inclusion in the clinical trials do not focus specifically at the target population that could be treated once the medicine goes on marketing authorization. For instance, many important trials on heart failure focused on a predominantly white male population having a mean age of ~60 years, whereas heart failure patients are actually older, more diverse, and have a higher mortality rate than the patients included in those trials (18). Similarly, underrepresentation of older patients has been reported in clinical trials of 15 different types of cancer (e.g., studies with only 25% of patients age 65 years and over, while the expected rate is >60%) (19). In this context, data from registries has been as a good regulatory tool used to fill these gaps for decision makers. For example, the FDA used the American Academy of Ophthalmology's intraocular lens registry to expand the label for intraocular lenses to younger patients. Registries may also be particularly useful for tracking effectiveness outcomes for a longer period than those usually realizable with clinical trials. For instance, some registries on growth hormone have tracked children up to the adulthood (14). One of the main principle of the AP regards the possibility to authorize a drug candidate for a small and well-defined patients populations in which the drug's benefits had clearly and already shown to outweigh its risk. This implies the setup of a collection of standardized information about a group of patients who share a condition or experience (20). In this way, the approval is expanded into new populations step by step, in an iterative fashion through the conduction of real-world trials setting in which the benefit/risk profile is continuously evaluated during the entire lifecycle of the medicine^12^. This new paradigm enables an additional opportunity for the companies to continuously gather clinical evidence about the medicine's benefits and risks from the real environment of use and setting. This strategy enable the possibility to receive earlier input from several stakeholders, especially from patients, prescribers, and payers and this could help to reduce costs and the risk of failures in an advanced stage of development (21). From an HTA point of view, this approach would allow applicants to place more emphasis on those groups of patients with the potentially higher “therapeutic value,” reducing the level of complexity of the evidence to be obtained. After this initial marketing authorization, the indication can be extended step by step after obtaining new evidence (4).

## The Main Characteristics of the Eligible Project for the AP Approach

There are a series of points to consider that could make the project eligible for the APs approach application, and that could satisfy some relevant expectations. As AP is a concept and not a procedure, the content of the proposal will determine whether the additional pre-submission meetings with applying companies can be granted. However, the design of a development plan that fulfill all the AP criteria has a greater degree of complexity, since it may encompass several indications, different subpopulations, and the design of protocols for data acquisition in the pre- and post- authorization phases (22). As previously reported, AP is based on three principles that should be taken into account. If not, other existing EMA processes are better suited to assist the applicant. During the pilot phase of the project, EMA received about 60 questions, 18 of which were selected for in-depth meetings, face to face with the participation of other interested parties. At the end of the pilot project, six of these applications had progressed to receive a formal parallel consultation from the EMA and HTA bodies and the others were assigned to traditional scientific advice. Most of the projects received for evaluation were deemed unsuitable for the AP and companies were advised to follow the standard development procedure. The pilot project has been functional to the evaluation of a series of aspects that need further reflections such as the increase in patient involvement, the definition of new strategies for collecting real data to support the evaluation of both efficacy and effectiveness and involvement of paying agencies and HTAs to provide input on pricing and reimbursement strategies on the basis of RWE. An important common denominator of all those procedures that were not considered suitable for the PA was the timing of the submission of projects for the AP approach. Often in fact the requests for development according to the AP approach were carried out too late while a fundamental prerequisite of the procedure is the early dialogue with all the relevant stakeholders involved (before the start of the phase II studies) in order to draw up a plan of intelligent development that allows a better acquisition of information from all possible data sources compared to the traditional RCT setting. A timeframe of a maximum of 6 months between the initial discussion and the submission of the advice request was strongly encouraged in order to finalize the advice request (23). After the process of evaluation that comprises the assessment of the prerequisites that meet the AP criteria, acceptance will be confirmed, in writing, to the company and EMA has consulted the stakeholders designated by the applicant to gauge their interest in discussing the proposal with the company and to participate to the additional pre-submission teleconference. Stakeholders may also choose to participate as observers (22).

## Challenges and the Opportunities Behind the APs Approach

Here we describe, on the one hand, the challenges and opportunities of the APs approach, analyzing specific issues that can allow us to optimize the timely access to medicine, and on the other hand, the problems for which the entire process still requires some improvements. Between several key aspects and actors that actively drive this process, the fulcrum is represented by patient demand for timely access and emphasis on unmet medical needs. This key aspect is highly influenced by other factors, such as emerging evidence with a fragmentation of treatment populations and early disease interception that is counterbalanced by the pressure and the need to optimize and to shorten all the regulatory procedures. Other main aspects include the pressure by Pharma industries and questions about sustainability and drug development which often require an extensive work by HTA opinion key leaders. One of the biggest challenges in a standard procedure, with the exceptions only for few orphan medicines and post-licensing safety studies, is to overcome the dogma represented by the need to obtain results exclusively from RCTs to be eligible for regulatory decisions. Information from other kind of studies is often considered not sufficiently acceptable by regulators and sometimes by payers (6, 24). However, this kind of approach often requires several years of development and the investment of huge resources with a high rate of failure (25). In the standard authorization process, the marketing authorization is often the primary goal of sponsors, while the procedures concerning the price and reimbursement authorization are addressed later and this is really dysfunctional, especially for some target population with an high unmet medical need. Following a standard authorization procedure, there is always a clear division between pre-and post-licensing; this criterium, in several situations—for example in the case of orphan diseases for which a limited number patients could be enrolled—can lead to a marketing approval of the medicine with a high level of uncertainty, particularly for such aspects concerning the safety and the efficacy, due to the intrinsic limit of feasibility of RCT for particular kinds of disease (26). Due to the intrinsic limits of tools and monitoring systems, there is a large gap between the actual difficulty in monitoring the real-data use of a drug. To date, also in view of the implementation of the regulation on pharmacovigilance, this kind of processes as well as different tools of early monitoring have been highly implemented. The request of high evidence standards from RCT in order to predict a drug's performance is not always feasible and necessary. This in light of the actual development of different diagnostic, screening, predictability, and pharmacogenomic tools that can allow the introduction of increased knowledge on the drug at an early stage of development. In fact, anticipated access implies, on the one hand, the need to accept a higher level of uncertainty and, on the other hand, the strengthening of cooperation between regulatory agencies, prescribers, patients, and payers to upgrade the production of evidence and to allow a more effective monitoring, in real time, for supporting decisions. Unfortunately, across Europe, there are still substantial differences between healthcare systems, because not all of them have robust tools to ensure that prescription of a medicine will only occur within the currently licensed population (27). These differences could lead in such countries with limited tools of monitoring to substantial and uncontrollable off-label use (28). This problem highlights the need to strengthen and standardize control instruments at a central level and to improve them in all those countries that still have limited resources. Another great obstacle regards the skepticism of conservative stakeholders. There is a general feeling that Science is moving faster than the regulatory systems, and this issue could be an obstacle for the right launch of the AP.

One important topic that laid the foundations of the AP, is the question whether standard approval procedures could still meet unsatisfied medical needs in a rapidly evolving scientific environment. Form this point of view the AP innovative approach, can guarantee the use of the most advanced discoveries and technologies available in the medical field to improve the quality of the evaluation process, reducing the number of redundant studies, optimizing the collection and use of clinical data, and reducing the delay between regulatory approval and patient/market access. Conversely, the AP has faced criticism from conservative experts for its perceived potential to lower safety standards and for the reliability of clinical trials in this new setting that deviates from the standard approaches^13^. However, is important to make an overview on different aspects that characterize this alleged lack of safety. Firstly, is important to specify that the primary role of the different experts involved in the process of evaluation is to assess the benefit-risk profile and not to guarantee the safety. The lack of effectiveness in late-stage development is the most frequent reason of clinical trial failures, and safety issues are more frequently observed after the marketing authorization, only when a larger number of patients have been treated. From this point of view, the challenge to the AP approach is to measure efficacy or effectiveness in a real world evidence setting with observational-like study designs, with the opportunity to assess the real acceptable level of uncertainty, also comparing it with the real clinical needs perceived by the patients (29). All these changes allow an “active monitoring” instead of a “passive prediction” and can facilitate a process toward a targeted prescription. Another advantage is represented by the fact that any potential positive benefit-risks assessed in a defined subpopulation add value to the drug, and can be followed by further clinical studies and trials in other sub-populations. This could lead to gradual enlargement (or limitation) of the label and the covered populations, as evidenced by recent data (6). With the adaptive pathway, the knowledge on the medicine increase not only since the first administration but also after the marketing approval, with the possibility of performing different cycles of learning-confirming-(re)licensing.

## The Importance of Parallel Scientific Advice and the Interaction With HTA Bodies

Since about 7 years, the procedures of parallel scientific advice have become an important discussion board for the establishment of rules and sharing requirements between regulators and HTA-bodies. The Parallel scientific advice procedures allow an harmonization between several different position from the HTA network, narrowing the gap between Regulators and HTA-bodies. This is an important topic because in the past years it was difficult to harmonize the regulatory requirements of the benefit-risk assessment with the different pricing and reimbursement criteria at national and regional level, with a delay on the authorization timing (30, 31)^14^. The parallel scientific advice allows the improvement of the predictability and transparency with a more harmonized selection of endpoints, comparators and with the increasing involvement of patients in the process of evaluation (30, 32). This would be such a great opportunity especially for medicines that address a high unmet medical need [e.g., within EMA's PRIME scheme] where there are often no clear clinical outcomes and endpoints for the new identified indication, but all the analyses were based exclusively through surrogate data. In order to evaluate the work of parallel scientific advice, between 2010 and 2015 EMA analyzed 31 procedures under the draft best practice guide. The results obtained showed a high level of commonality in the evidence requirements between participating decision-makers on issues like active comparators, clinical endpoints, and outcomes (33). Actually there is a strict collaboration and coordination between EMA and EUnetHTA in order to provide indication to simultaneous and coordinated advices in the development projects of medicines, with a view to facilitate the alignment of data from the initial evidence generation to post-authorization data collection. The goal of these coordinated actions is to generate an efficient environment of evidences that could satisfy the criteria, the rules, and the expectations from regulators and HTA bodies. The need of the EMA to work with all decision-makers can ensure that medicines potentially making a real difference for health can actually reach the patient earlier. In order to improve the process of evaluation and to ensure high-quality advice and consistency over time, EUnetHTA created the Early Dialogues Working Party (EDWP), that comprises HTA bodies having demonstrated experience in Early Dialogues/Scientific Advice. This platform can increase the mutual understanding and problem-solving ability through a more structured interaction an improved coordination between EMA and HTA bodies^15^^,^^16^.

## Conclusions and Perspectives

Early access tools aim to get new promising medicines for patients more quickly than is currently possible. However, providing patients with timely access to new medicines increases uncertainty about the risk-benefit profiles of these products. The concept of AP must be interpreted precisely in order to overcome the limits that were found in the use of these tools. AP fits precisely in this context: to favor and increase an early dialogue among all relevant stakeholders in the medicine evaluation process and to increase the use of all available and useful tools for a more efficient collection of all useful information, based on the existing regulatory framework. The use of early access tools has in fact proved to be inefficient in all those cases in which requests for access to these tools were made too late when the margin for using corrective measures was now impossible or reduced to a minimum. Obviously finding a balance between early access and the need to obtain certain data requires a greater effort on the part of the regulatory authorities or greater post-marketing surveillance and a continuous assessment of the risk-benefit ratio of the product during the various phases of experimentation clinic (34, 35). In this perspective, the primary objective of EMA concerns the optimization of all regulatory instruments developed in line with the existing regulatory framework. The type of marketing authorization obtained, including any conditional approval or approval under exceptional circumstances, must be determined case by case, based on the level of evidence obtained^17^. In this context the AP approach must be considered a valid opportunity to foster a fruitful early dialogue between all interested parties (regulators, HTA bodies, and patient representatives). The primary purpose of early dialogue is to explore ways to optimize drug development pathways with the potential goal of accelerating patient access to drugs and optimizing data collection in all those contexts where standard authorization procedures they are not optimal or easy to implement (i.e., orphan drugs, medicine with high unmet medical need, etc.). To achieve these objectives, the pilot project also analyzed alternative tools that could reduce the time between the marketing authorization and the marketing and reimbursement phases; in this perspective, for example, the early involvement of HTA bodies and payers is of fundamental importance. Due to the nature of APs, only products that meet unmet medical needs and are at an early stage of clinical development are eligible for inclusion in the AP scheme. In particular, in this context, “initial stage” means all the phases preceding the beginning of the confirmation studies, before the beginning of the Phase II clinical phase. Inclusion in the initial phase allows a significant and effective contribution by all interested parties on development planning, licensing, monitoring, reimbursement, and consistent use paths^18^. The relative unfamiliarity with APs among stakeholders also fuels the idea that APs are “the last resort” for medicine manufacturers who may not easily comply with the standard authorization criteria. For some of the parties involved, this impression appeared to be supported by the fact that some manufacturers have proposed, for inclusion in the AP scheme, products that did not meet the selection criteria, or were in the middle of the normal authorization process, or were high risk of not obtaining regular marketing authorizations. In any case, the APs approach represents an opportunity for drug development, allowing an ever greater level of transparency between industry, decision-makers, and patients who use the new medicines. In this context, the involvement of regulatory authorities in the risk monitoring and mitigation phase from the initial licensing process remains essential. This can be achieved through a most widespread implementation of the registers, the use of data collected through electronic tools (i.e., electronic medical records) and the development of post-authorization observational studies. Recently the EMA has issued a strategic reflection document with a vision of perspective to 2025 which highlights issues of extreme importance. As EMA's Executive Director says “*the peace of innovation has undergone a dramatic acceleration in recent years and regulatory bodies must be ready to support the development of more complex drugs that offer more and more health solutions.”* The document focuses on important issues such as the need to catalyze the integration of science and technology in medicines development promoting and investing in the PRIME scheme, driving collaborative evidence generation toward the improvement of the scientific quality of evaluations. One point that we consider with particular interest, from our point of view, regards the need to advance patient-centered access to medicines in partnership with healthcare system: contributing to HTA's preparedness and downstream decision making for innovative medicines; Bridging from evaluation to access through collaboration with payers. All these aspects reinforce significantly the patient relevance in evidence generation and are the basis for the development of a solid network of competences and specialist collaborations to engage with the use of big data^19^. The EMA is greatly re-evaluating the importance of the effective contribution of early dialogue with all the stakeholders involved in its initiatives since, obtaining feedback and the debate opened by and with the interested parties, prove to be increasingly essential for starting better and more efficient drug development programs with a significant reduction in uncertainty margins, while improving the benefit-risk ratio even in those settings where the use of standard authorization procedures is difficult or not applicable. In conclusion, is important to remember that the adaptive pathway approach is a concept still in development and for this reasons requires a fine-tuning as more medicine in development are included to be eligible for, especially in situation where the classical clinical trial design approach is not feasibly applicable or too lengthy; in this kind of situation a novel approach to evidence generation and decision-making might lead to accelerated patient access.

## Author Contributions

GN, GS, and PP substantially contributed for the conception and design of the review. AS contributed substantially in drafting and revising the review article critically ensuring that questions related to the accuracy or integrity of any part of the work are appropriately investigated and resolved.

### Conflict of Interest Statement

The authors declare that the research was conducted in the absence of any commercial or financial relationships that could be construed as a potential conflict of interest.

## Footnotes

^1^*Accelerated Development of Appropriate Patient Therapies a Sustainable, Multi-Stakeholder Approach from Research to Treatment-Outcomes*. Available online at: http://adaptsmart.eu/addressing-drivers-opportunities-and-obstacles-of-mapps/ (accessed January 2019).

^2^https://www.ema.europa.eu/en/human-regulatory/research-development/adaptive-pathways (accessed April 2019).

^3^https://www.ema.europa.eu/en/documents/regulatory-procedural-guideline/european-medicines-agency-guidance-applicants-seeking-scientific-advice-protocol-assistance_en.pdf (accessed April 2019).

^4^https://www.ema.europa.eu/en/documents/regulatory-procedural-guideline/guidance-parallel-consultation_en.pdf (accessed April 2019).

^5^https://www.ema.europa.eu/en/documents/other/general-principles-european-medicines-agency-food-drug-administration-parallel-scientific-advice_en.pdf (accessed April 2019).

^6^https://www.ema.europa.eu/en/documents/regulatory-procedural-guideline/enhanced-early-dialogue-facilitate-accelerated-assessment-priority-medicines-prime_en.pdf (accessed April 2019).

^7^https://www.ema.europa.eu/en/documents/scientific-guideline/guideline-scientific-application-practical-arrangements-necessary-implement-commission-regulation-ec/2006-conditional-marketing-authorisation-medicinal-products-human-use-falling_en.pdf (accessed April 2019).

^8^https://ec.europa.eu/health//sites/health/files/files/eudralex/vol-1/reg_2004_726/reg_2004_726_en.pdf (accessed April 2019).

^9^https://www.ema.europa.eu/en/documents/scientific-guideline/guideline-scientific-application-practical-arrangements-necessary-implement-procedure-accelerated/2004_en.pdf (accessed April 2019).

^10^*European Medicines Agency Final report on the APs pilot*. EMA/276376/2016.

^11^*Spinraza Drug Authorization: Patients' Organization AFM-Telethon Welcomes a Major Step but Calls Upon Full Transparency by Biogen Regarding the Price Claimed*. Available online at: http://www.afm-telethon.com/news/spinraza-drug-authorization-patients-organization-afm-telethon-welcomes-a-major-step-but-calls-upon-full-transparency-by-biogen-regarding-the-price-claimed.html (accessed April 2018).

^12^*All Eyes on EMA's Adaptive Licensing Pilot*. http://www.centerwatch.com/news-online/2015/01/01/all-eyes-on-emas-adaptive-licensing-pilot/ (accessed July 2018).

^13^*EMA Returns to the Fray on APs*. Available online at: http://www.pharmexec.com/ema-returns-fray-adaptive-pathways (accessed January 2018).

^14^*Early Dialogue with Regulators and HTA Bodies*. EMA/187274/2016 (2016). Available online at: http://www.ema.europa.eu/docs/en_GB/document_library/Press_release/2016/03/WC50203950.pdf. (accessed May 2018).

^15^*Parallel Consultation with Regulators and Health Technology Assessment Bodies*. Available online at: https://www.ema.europa.eu/en/human-regulatory/research-development/scientific-advice-protocol-assistance/parallel-consultation-regulators-health-technology-assessment-bodies (accessed September 2018).

^16^EMA/390765/2017 Media and Public Relations; Press release; EMA and EUnetHTA step up interaction to align data requirements.

^17^EMA/417706/2014. Human Medicines Research and Development Support: Questions and answers following the initial experience of the Adaptive Licensing Pilot project.

^18^*The Adaptive Pathways Process: Benefits and Potential Hurdles from a Dutch Perspective RIVM Report 2016-0102*. Available online at: http://www.rivm.nl/dsresource?objectid=473c1314-ba06-4b77-819c-bd6ccb6dca9b&type=org&disposition=inline (accessed July 2018).

^19^EMA Regulatory Science to 2025. Strategic reflection. Available online at: https://www.ema.europa.eu/documents/regulatory-procedural-guideline/ema-regulatory-science-2025-strategic-reflection_en.pdf (accessed February 2019).

## Abbreviations

EMA: European Medicines Agency
AP: Adaptive Pathways
HTA: Heath Technology Assessment
FDA: Food and Drug Administration
RCT: Randomized Controlled Trial
EUPATI: European Patients Academy on Therapeutic Innovation'
SMA: Spinal Muscular Atrophy
AFM-Téléthon: French Muscular Dystrophy Association
TuA: Temporary use Authorizations
RWE: Real World Evidence
PRIME: PRIority MEdicines
EUnetHTA: European Network for Health Technology Assessment
EDWP: Early Dialogues Working Party.
